# Mapping small metabolite changes after traumatic brain injury using AP-MALDI MSI

**DOI:** 10.1007/s00216-024-05422-6

**Published:** 2024-08-01

**Authors:** Angela Marika Siciliano, Federico Moro, Giulia De Simone, Francesca Pischiutta, Aurelia Morabito, Roberta Pastorelli, Laura Brunelli, Elisa R. Zanier, Enrico Davoli

**Affiliations:** 1 Mass Spectrometry Research Centre for Health and Environment and Laboratory of Mass Spectrometry, Environmental Health Sciences Department, Istituto di Ricerche Farmacologiche Mario Negri IRCCS, 20156 Milan, Italy; 2 Laboratory of Traumatic Brain Injury and Neuroprotection, Department of Acute Brain and Cardiovascular Injury, Istituto di Ricerche Farmacologiche Mario Negri IRCCS, Milan, Italy; 3 Laboratory of Protein and Metabolites in Translational Research, Environmental Health Sciences Department, Istituto di Ricerche Farmacologiche Mario Negri IRCCS, 20156 Milan, Italy; 4 Department of Electronics, Information and Bioengineering, Politecnico di Milano, 20133 Milan, Italy

**Keywords:** Traumatic brain injury, Small metabolites, AP-MALDI, Mass spectrometry imaging

## Abstract

Traumatic brain injury (TBI) is an alteration of brain function caused by a sudden transmission of an external force to the head. The biomechanical impact induces acute and chronic metabolic changes that highly contribute to injury evolution and outcome. TBI heterogeneity calls for approaches allowing the mapping of regional molecular and metabolic changes underpinning disease progression, with mass spectrometry imaging (MSI) as an efficient tool to study the spatial distribution of small metabolites. In this study, we applied an innovative targeted atmospheric pressure-MALDI mass spectrometry imaging (AP-MALDI MSI) approach, starting from an extensive list of metabolites, representative of different metabolic pathways, individually validated on the tissue under analysis with original standards using 2,5-dihydroxybenzoic acid (DHB), to characterize the impact of TBI on regional changes to small metabolites in the brain. Brains from sham and TBI mice obtained 21 days post-injury were analyzed to examine the spatial metabolic profile of small metabolites belonging to different metabolic pathways. By a whole brain analysis, we identified four metabolites (alanine, lysine, histidine, and inosine) with higher abundance in TBI than sham mice. Within the TBI group, lysine, histidine, and inosine were higher in the hemisphere ipsilateral to the biomechanical impact vs. the contralateral one. Images showed a major involvement of the ipsilateral thalamus characterized by the increase of arginine, lysine, histidine, and inosine and a significant reduction of glutamic acid, and N-acetylaspartic acid compared to the contralateral thalamus. These findings indicate high-resolution imaging mass spectrometry as a powerful tool to identify region-specific changes after a TBI to understand the metabolic changes underlying brain injury evolution.

**Supplementary Information:**

The online version contains supplementary material available at 10.1007/s00216-024-05422-6.

## Introduction

Traumatic brain injury (TBI) is a leading cause of death and disability, with 60 million people suffering from a TBI each year [1] and no neuroprotective or regenerative treatments available. Experimental models of TBI are valuable tools for mimicking key clinical neuropathological features of TBI patients, including behavioral deficits, progressive neurodegeneration, white matter damage, and neuroinflammation and the development of post-traumatic epilepsy [2–6]. After TBI, changes in metabolites, such as amino acids, fatty acids, and acylcarnitines, can sustain cytotoxicity, oxidative stress, and neuroinflammatory processes [6, 7] but their spatial characterization with imaging approaches is lacking. Various techniques have been employed to map the distribution of small molecules in specific areas, aiding in the discovery and study of metabolic changes at the local level. While magnetic resonance spectroscopy (MRS) can identify in vivo metabolic alterations after brain injury [8, 9], its limited spatial resolution restricts its ability to detect less abundant metabolites and discriminate molecules with similar molecular weights. In recent years, mass spectrometry imaging (MSI), and specifically matrix-assisted laser desorption/ionization (MALDI) MSI, has gained prominence [10]. This technique, involving the application of specific matrices onto biological tissue sections, enables the direct visualization of molecules with varying natures (such as proteins, lipids, endogenous metabolites, and drugs) with high sensitivity, specificity, and spatial resolution [11–14].

In recent years, MALDI MSI has been coupled with atmospheric pressure ionization (AP)-MALDI MSI offering several advantages, like avoiding desiccation to prevent tissue cracking or analyte crystallization, the advantage of improved spatial analyte identification, and, mainly, the advantage of improved spatial analyte identification. This is attributed to the elimination of the need for vacuum and drying processes in samples [15]. Despite its potential as a discovery tool for the detection of small molecules over biological tissue, there have been limited studies utilizing this technique in the context of experimental TBI, with no studies available in mice [7, 16, 17].

In this study, we employed targeted AP-MALDI MSI to assess spatial changes in brain metabolites in mice with traumatic brain injury (TBI). Our investigation specifically concentrated on discerning spatial alterations in metabolites following TBI, enabling us to describe sub-acute modifications in the brain that may contribute to the evolution of the injury.

## Material and methods

### Animals

Eight-week-old CD1 male mice (~ 25–30 g; Envigo, Italy) were maintained in SPF facilities and housed at constant room temperature (23 °C) and relative humidity (60 ± 5%) with free access to food and water and a fixed 12-h light/dark cycle. Mice were individually housed with environmental enrichment. Procedures involving animals and their care were conducted in conformity with the institutional guidelines at the Istituto di Ricerche Farmacologiche Mario Negri IRCCS in compliance with national standards (D.lgs 26/2014; Authorization n. 19/2008-A issued March 6, 2008, by Ministry of Health); Mario Negri Institutional Regulations and Policies providing internal authorization for persons conducting animal experiments (Quality Management System Certificate – UNI EN ISO 9001:2015 – Reg. N° 6121); the NIH Guide for the Care and Use of Laboratory Animals (2011 edition); and EU directives and guidelines (EEC Council Directive 2010/63/UE). All animal experiments were designed in accordance with Animal Research: Reporting of In Vivo Experiments (ARRIVE) guidelines [18], with a commitment to refinement, reduction, and replacement, and using biostatistics to optimize the number of mice.

### TBI animal’s model

Mice (*n* = 6) were analyzed with isoflurane inhalation (2% for induction, 1.5% for maintenance) in an N_2_O/O_2_ (70%/30%) mixture and placed in a stereotaxic frame, then subjected to craniectomy followed by induction of CCI brain injury as previously described [19, 20]. Briefly, the injury was induced using a 3-mm-diameter rigid impactor driven by a pneumatic piston rigidly mounted at an angle of 20° from the vertical plane and applied perpendicularly to the exposed dura mater, between bregma and lambda, over the left parieto-temporal cortex (antero-posteriority: − 2.5 mm, laterality: left 2.5 mm), at an impactor velocity of 5 m/s and deformation depth of 2 mm, resulting in severe injury [19, 20]. The craniectomy was then covered with a cranioplasty and the scalp sutured. Control sham mice (*n* = 6) received identical anesthesia, and surgery, but no brain trauma. During all surgical procedures, mice were maintained at a body temperature of 37 °C.

### Tissue sectioning for MALDI MS imaging

Slides of serial mice brain section of 10 µm from frozen brains were cut using a cryo-microtome (Leica Microsystems, Wetzler, Germany) at − 20 °C and directly placed on a pre-cooled Opti-TOF insert (SCIEX®, Italy; No. 4347684) for instrumental analysis. The adjacent coronal brain slices (10-µm thickness) were collected for subsequent immunohistochemical staining, mounted on a POLYSINE™ microscope slide (VWR, Belgium; cat. no. 631-0107), and stored at − 20 °C [21].

Before matrix application, the Opti-TOF insert with brain slides was placed into a vacuum desiccator overnight. Hematoxylin and eosin (H&E) staining was performed on adjacent tissue slides as reported in the literature [22].

### Matrix application and MALDI MS imaging

DHB matrix was chosen because it provided the best results on the panel of targeted metabolites used [23]. The AP-MALDI imaging experiment was conducted using 2,5-dihydroxybenzoic acid (DHB) (MilliporeSigma, Germany). The matrix solution (DHB, 30 mg/ml methanol 80%) was sprayed on brain slices using SunCollect sprayer (SunChrom®, Germany) following the methods: spray speed 800 mm/min, line distance 2 mm, 12 layers (1, 10 µl/min; 2, 20 µl/min; 3, 30 µl/min; 4, 50 µl/min, from 5 to 12: 60 µl/min). The sprayed slide was dried at room temperature for 30 min until analysis.

### AP-MALDI high-resolution mass spectrometry imaging and data analysis

Mass spectrometry imaging was performed using an AP-MALDI ultra high-resolution source (MassTech Inc., MD, USA) mounted on an Orbitrap Q-Exactive instrument Q-Exactive (Thermo Scientific®, MA, USA) mass spectrometer. The AP-MALDI ion source was equipped with a diode-pumped solid-state laser (*λ* = 355 nm) operating at a 0.1–10 kHz repetition rate. The experiments were performed using Target-ng software (MassTech Inc., MD, USA) and the source operating in continuous motion mode. The laser was set to 5 kHz with laser energy of 15% and 100 µm of imaging spatial resolution. Plate velocity of 7.4 mm min^−1^ with pixel duration of 0.81 s were used as imaging parameter acquisition. The mass spectrometer method on Q-Exactive instrument was set using Tune (Thermo Scientific, Bremen, Germany) in Full MS mode. The acquisition range of 50–500 m/*z* was used in the positive polarity for the DHB matrix. The resolution was optimized to 70,000 and automatic gain control (AGC target value) of 5e6 with maximum ion injection time of 500 ms, while the capillary temperature was set to 320 °C. The AGC target value and maximum ion injection time were optimized using a tissue control for the matrix.

The mass calibration was executed using ESI source mounted on the Q-Exactive instrument using Pierce™ ESI negative ion calibration solution (Thermo Scientific, Bremen, Germany; cat. no. 88324) and Pierce™ LTQ Velos ESI positive ion calibration solution (Thermo Scientific, Bremen, Germany; cat. no. 88323) before the AP-MALDI imaging acquisition. The imaging data obtained were processed using ImageQuest (Thermo Scientific, Bremen, Germany) and MSiReader (v 1.02; NC State University, Raleigh, NC) applying a local TIC normalization with 6 ppm tolerance for the ion images generation (Robichaud et al., 2013). The region of interest (ROI) was constructed manually on each image through MSiReader using a histological staining (H&E) as guide to identify specific areas.

### Metabolite identification

This work is based on a targeted approach on positively confirmed compounds only, in order to identify changes even for small metabolites. We started from an extensive list of individual metabolites, belonging to main metabolic pathways [24] and we optimized the targeted acquisition. The MS/MS experiments were conducted on tissue slices before the AP-MALDI experiment to validate the presence of each metabolite in the brain tissue. Each individual metabolite original standard has been validated directly on-tissue. Human Metabolome Database (HMDB) was used to confirm metabolite identity. Xcalibur (Thermo Scientific, Bremen, Germany) was used to set the MS/MS fragmentation with normalized collision energy (NCE) of 15 and an isolation window of 3 m/*z*. An inclusion list of selected ions corresponding to the metabolites searched was used in a data-independent acquisition (DIA) mode MS/MS. The obtained fragment was put into the HMDB and the identity was confirmed excluding the matrix interference. For each identified metabolite, the images were constructed from the specific *m/z*. The distribution of each metabolite was assessed with a spatial resolution of 100 µm using relative abundances, normalized to the area of each slice. Local TIC was applied to normalize the tissue slices and images are created with the same intensity scale for each metabolite.

### LC-MS validation

For LC/MS analysis, two adjacent 20-µm-thick sections were collected into an Eppendorf tube of 50 µl pre-cooled at − 20 °C and stored at − 80 °C until the analysis. Metabolites from slices were extracted using 50 µl of extraction solvent (50:30:20 MeOH/ACN/H_2_O), homogenized using a pestle motor mixer (Argos), incubated for 20 min at − 80 °C, and centrifuged at 13,000 g × 15 min. Supernatant were stored at − 80° C until the metabolomics analysis.

The LC/MS-MS approach investigated the abundance of metabolites by using liquid chromatography coupled with a triple quadrupole mass spectrometry system (LCMS-8060, Shimadzu). Five (5) µl of metabolite extract was injected into a Discovery HS F5-3 (2.1 mm I.D. × 150 mm, 3 µm) column (Sigma-Aldrich), using a 20-min gradient from 0 to 95% B (acetonitrile) and A (10 mM NH₄HCO₂ pH 3.5) at 350 µl/min. The LCMS-8060 mass spectrometer (Shimadzu, Kyoto, Japan) was equipped with an ESI source operating in positive ion and selected reaction monitoring (SRM) mode. The transitions identified during the optimization of the method are listed in the Supplementary Table S2. The MS settings were as follows: nebulizing gas flow rate, 3.0 l/min; drying gas flow rate, 15.0 l/min; DL temperature, 250 °C; block heater temperature, 400 °C. Peak areas were automatically integrated using LabSolution Insight LC MS (Shimadzu, Kyoto, Japan). Retention times and area ratio between quantifier and qualifier ions of all metabolites of interest were validated using pure standards (Sigma-Aldrich). The metabolites were extracted from all the biological independent replicates from the slice’s homogenates collected after sampling for the MSI analysis, both for sham and TBI conditions.

### Statistical analysis

Data normality was assessed by the Kolmogorov-Smirnov test. Data was considered normal if *p* > 0.05. Since most data followed a normal distribution, significant differences between the two groups were assessed by unpaired *t*-test with significance cutoff *p* < 0.05. All analyses were performed using GraphPad Prism software version 9.3.1

## Results

### Targeted AP-MALDI MSI strategy identifies changes of small metabolites

**Fig. 1:**
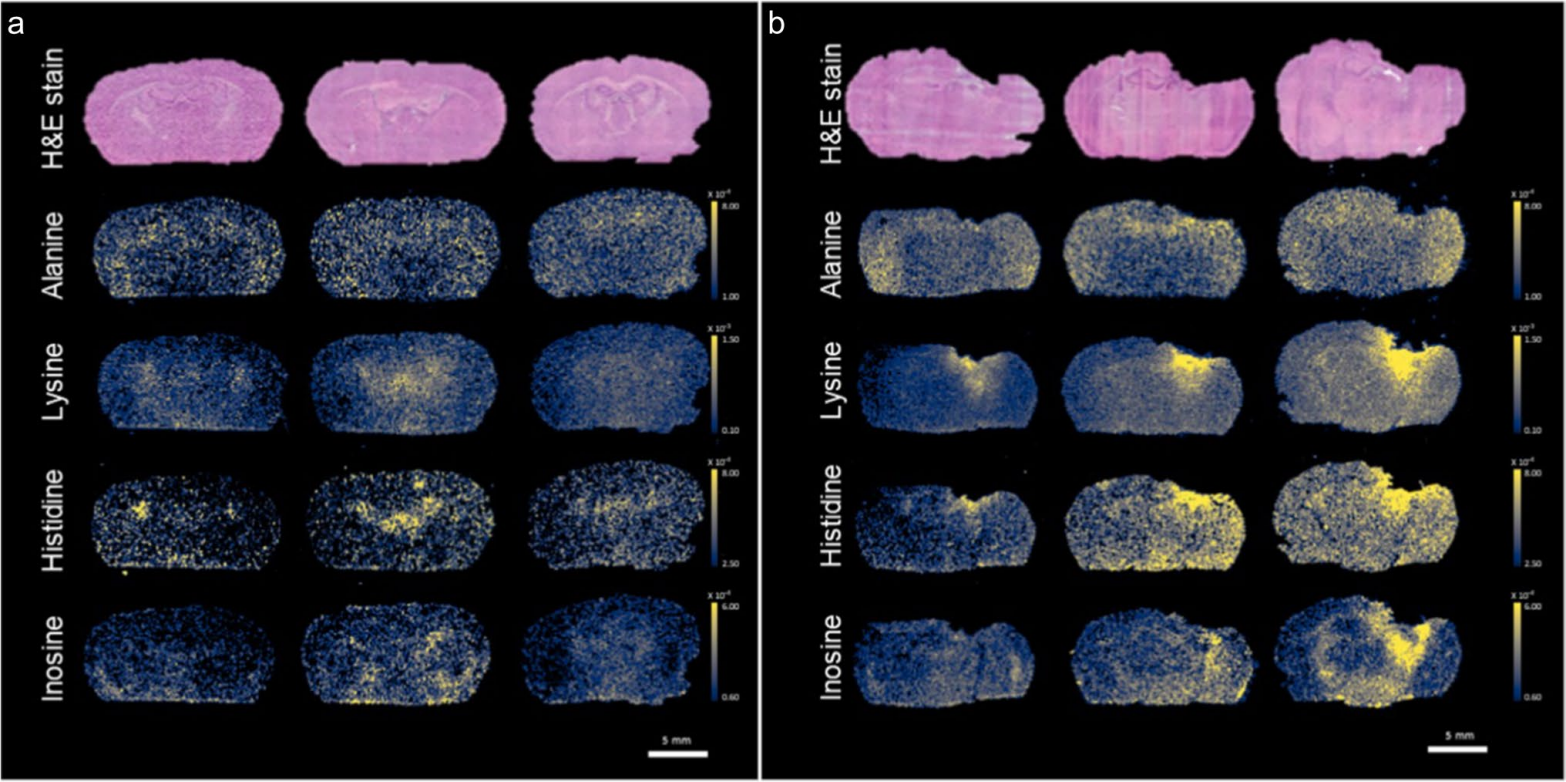
Representative images of metabolite distribution in sham and TBI mice, 21 days post-injury. Hematoxylin/eosin (H&E) staining and the spatial distribution of alanine, lysine, histidine, and inosine in the three representative biological replicates of sham (**a**) and TBI (**b**) brains at 21 days. Scale bar = 5 mm. Cividis black color scale indicates ion intensity

Alteration in the spatial distribution of small metabolites in mouse brain after TBI (six replicate/condition) was analyzed 21 days post-injury. Based on the tandem mass spectrometry (MS/MS) approach, we reconstructed the spatial distribution of 23 metabolites listed in Supplementary Table S1 and showed in Supplementary Figure S1. Among these, alanine, lysine, histidine, and inosine showed a drastic difference of distribution between sham and TBI (Fig. 1).

**Fig. 2:**
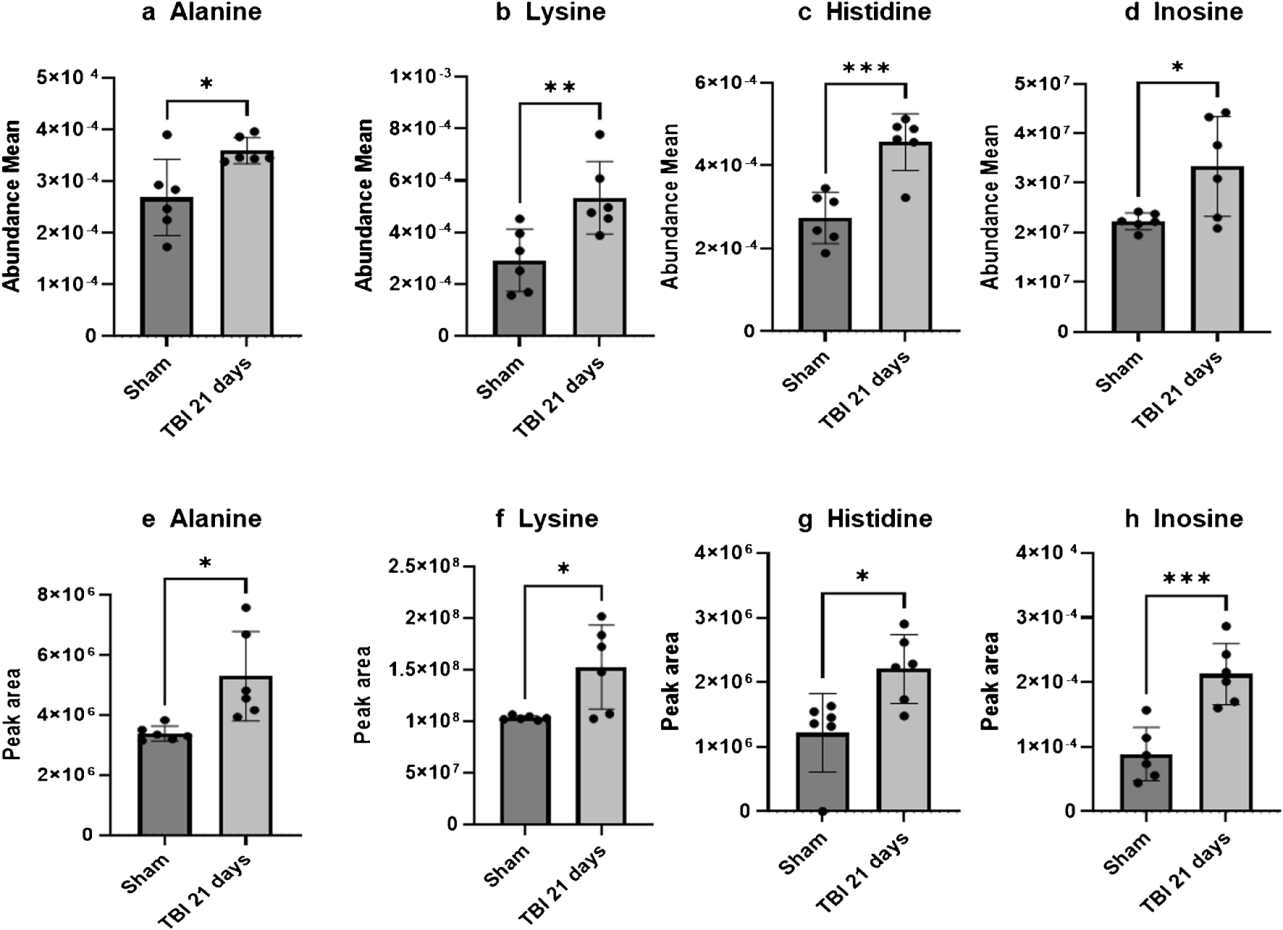
Whole brain ion abundances of metabolites with different amounts in TBI vs. sham brains. Brain amounts of alanine, lysine, histidine, and inosine performed by MSI analysis (**a**–**d**) or by LC-MS analysis (**e**–**h**). Data are shown as mean ± SD, *n* = 6, unpaired *t*-test *p < 0.05, **p < 0.01, ***p < 0.005

By considering the whole brain slice ion abundances, a significant higher amount of alanine, lysin, histidine, and inosine in TBI compared to sham was detected (Fig. 2a–d). To confirm the metabolic changes obtained by AP-MALDI MSI, we validated the results by using a targeted liquid chromatography-mass spectrometry (LC-MS) method on homogenized adjacent brain slices. Targeted LC-MS analyses confirmed the increase of alanine, lysine, histidine, and inosine in TBI vs. sham brains (Fig. 2e–h).

**Fig. 3:**
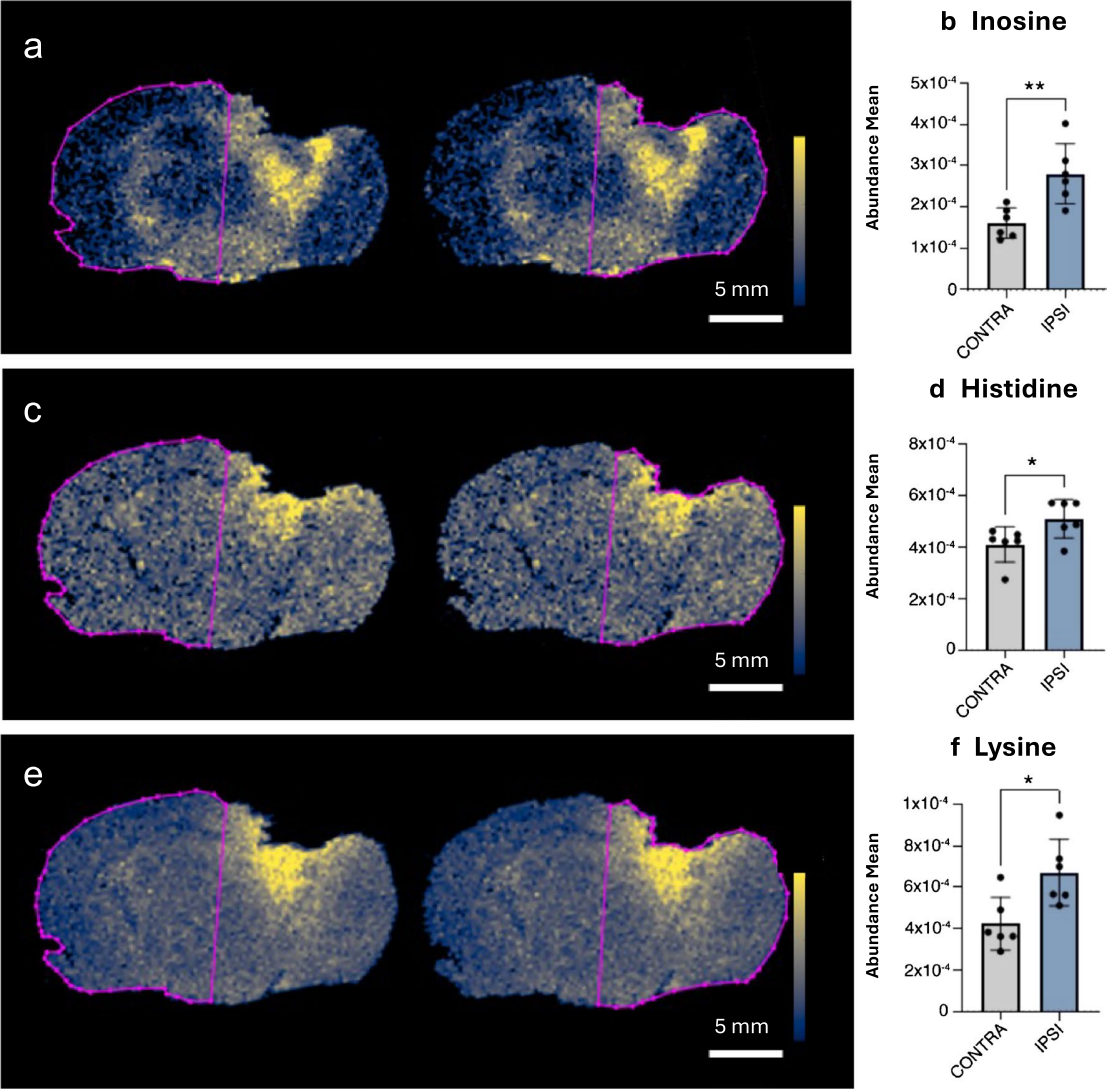
ROI-based ion abundances of metabolites in TBI ipsi- vs. contralateral hemispheres. Images showing the spatial distribution of inosine, histidine, and lysine in contralateral vs. ipsilateral hemisphere (**a**, **c**, **e**) by MSI and their relative ion abundances (**b**, **d**, **f**). Scale bar = 5 mm. Cividis black color scale indicates ion intensity. Data are shown as mean ± SD, *n* = 6, unpaired *t*-test *p < 0.05, **p < 0.01

Spatial images analysis showed an overall increase in the metabolite levels in the ipsilateral brain area adjacent to the contusion; thus, we assessed differences between ipsi- and contralateral hemispheres by manually drawing two ROI based on hematoxylin and eosin (H&E) staining. TBI mice showed a significant increase in ipsi- vs. contralateral hemisphere in three out of four metabolites identified in the whole brain (inosine, histidine, and lysine). These findings indicate that, as expected, the injured hemisphere is the area most affected by metabolite alterations (Fig. 3).

### From whole brain to thalamic region: a focus on the metabolite’s spatial changes

**Fig. 4:**
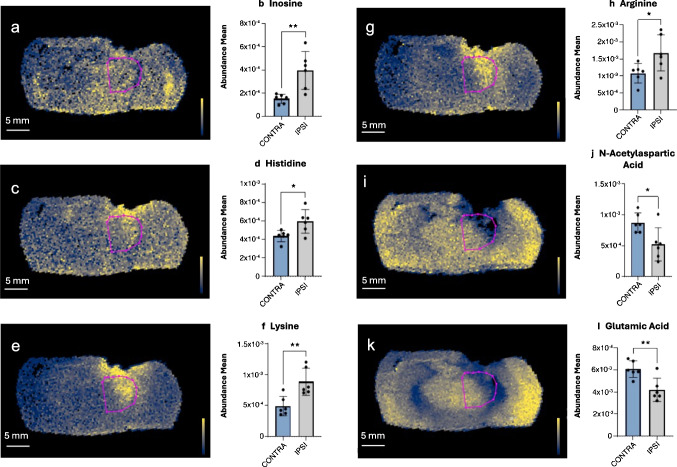
ROI-based analysis of thalamic regions. Images show the spatial distribution of inosine (**a**), histidine (**c**), lysine (**e**), arginine (**g**), N-acetylaspartic acid (**i**), and glutamic acid (**k**) in contralateral vs. ipsilateral thalamic regions by MSI and their relative ion abundances (**b**, **d**, **f**, **h**, **j**, **l**). Scale bar = 5 mm. Cividis black color scale indicates ion intensity. Data are shown as mean ± SD, *n* = 6, unpaired *t*-test *p < 0.05, **p < 0.01

Since the highest metabolic changes appeared to be mainly localized at the level of the ipsilateral thalamus, we further investigated the thalamic region with a ROI-based approach. Through the employment of MSI, it was actually possible to identify which brain area was most affected by the changes in the identified metabolites, giving us a powerful tool to focus the analysis in a specific brain region. Importantly, the thalamus is a brain area deeply impacted in our TBI model and linked to an unfavorable outcome following brain injury [22, 23]; additionally, considering the distribution of the identified metabolites in the whole brain, the thalamus visually appeared to be the most affected area, being linked to an increase in the relative abundance of the examined metabolites. We thus drew ROI over thalamic regions to identify changes of metabolic amounts. Ion abundances confirmed an increased amount of the three previously identified metabolites that increased in the ipsilateral hemisphere, inosine, histidine, and lysine (Fig. 4a–f). In addition, we detected a significant increase of arginine (Fig. 4g, h) and a decrease in glutamic acid and N-acetylaspartic acid (NAA) (Fig. 4i–l).

**Fig. 5:**
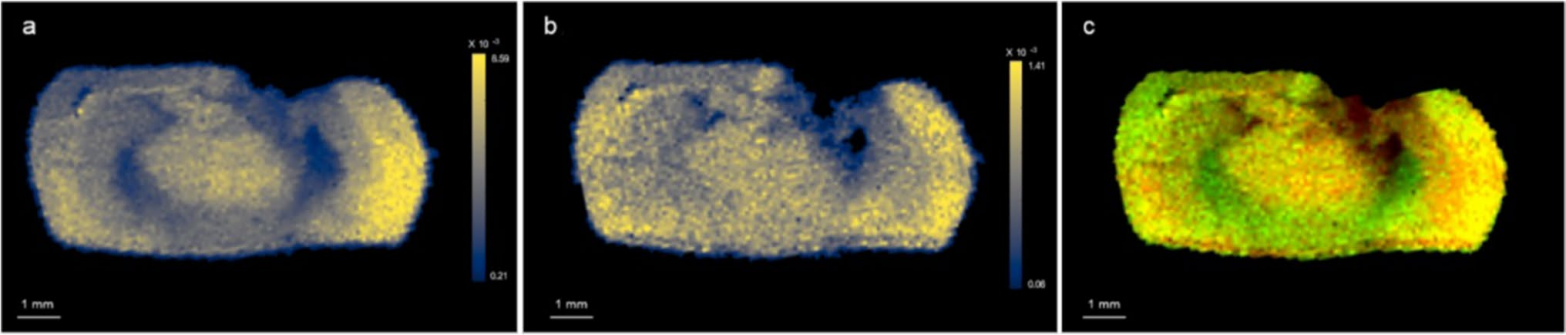
Co-localization of glutamic acid and N-acetylaspartic acid of TBI brain 21 days post-injury. **a** Spatial distribution of glutamic acid (*m*/*z* 148.0553) and **b** N-acetylaspartic acid (*m*/*z* 176.0553) and **c** co-localization of glutamic acid (red color) and N-acetylaspartic acid (green color). Co-localization of decreased metabolites is reflected by yellow color better highlighting the distribution of these two metabolites in the coronal brain section

Furthermore, by the co-localization of glutamic acid and N-acetylaspartic acid, the decrease in glutamic acid and N-acetylaspartic acid was highlighted spatially, as shown in Fig. 5.

## Discussion

Using the innovative targeted AP-MALDI MSI approach, we mapped the distribution of small validated metabolite changes after TBI in mice. We characterized the spatial distribution of the increased levels of three amino acids (l-lysine, histidine, alanine) and a nucleoside (inosine), comparing the sham and TBI condition 21 days after injury that may contribute to alteration in brain functionality. It has been documented that l-lysine is catabolized through two separate pathways, the saccharopin and the pipecolate, both converging in a common degradative pathway [25]. In the adult brain, the pipecolate pathway leading to l-pipecolate formation predominates, whereas in extracerebral tissues, the pipecolate pathway is a minor pathway for lysine degradation [14]. We showed that the alteration of the distribution of this metabolite can be identified in the injured area, but future studies are required to understand how and whether this impairment might affect the metabolite degradation pathway. The second amino acid found elevated 21 days after TBI was histidine, an essential amino acid acquired from the diet. It works as a precursor to carnosine in human muscle and some brain regions, as well as to histamine via decarboxylation [26]. The latest altered amino acid, alanine, showed a similar pattern of l-lysine and histidine with an increase in TBI mice. Notably, unlike lysine and histidine, showing increased concentrations in the pericontusional areas, alanine showed an increase throughout the brain section, both in ipsilateral and contralateral TBI cortex. Alanine is among the naturally occurring amino acids found in mammals [27]. In the brain, it has a key role in regulating neurotransmitter levels and it is involved in energy metabolism. It can be converted to pyruvate, an important molecule for energy production through the process of glycolysis. From this evidence, its role has been investigated in different pathological conditions, including TBI. Amorini and colleagues [28] demonstrated an increase in alanine concentrations up to 48 h after severe TBI in whole brain extract, a difference that was not present anymore at 5 days. Our AP-MALDI MSI–based approach allows for a more precise regional quantification, showing increased alanine levels up to 21 days after injury highlighting that MSI strategy could be effectively used to map the distribution of metabolic changes at the sub-chronic stage after experimental TBI in mice. The last increased metabolite identified was inosine. Inosine has been discovered to act as a signalling molecule in various conditions such as inflammation, brain injury, or ischemia [29]. The study by Michiel J. Bell and colleagues [30] has reported the crucial role of inosine in TBI, revealing an increase in inosine concentrations in the cerebral interstitial fluid of rats following controlled cortical impact. Our findings suggest that inosine levels remain significantly impaired in the ipsilateral thalamus even 21 days after TBI. These results might indicate an impairment of the physiological metabolic pathways of adenosine resulting from excessive energy expenditure in the thalamus. It is noteworthy that the thalamus serves as a propagation point in the epileptogenic network [31]. Additionally, inosine levels play a role in regulating the pathomechanisms of epilepsy [32]. This observation suggests that inosine levels in the thalamus may modulate the development of seizures following traumatic brain injury (TBI).

Importantly, AP-MALDI MSI enabled the identification of specific reductions in the levels of arginine, glutamic acid, and N-acetylaspartic acid (NAA) in the ipsilateral thalamus. Notably, these reductions had not been previously detected through hemisphere analysis. Additionally, our findings confirmed that the observed increase in lysine, histidine, and inosine was also specific to the ipsilateral thalamus.

l-Arginine (Arg) is a semi-essential amino acid that can be enzymatically metabolized to produce a number of biologically active molecules. Changes in Arg levels in the brain can lead to increased nitric oxide production, resulting in neurotoxicity and neurodegeneration [33]. The elevated Arg levels in the thalamus 21 days after TBI, suggest the persistence of long-term neurotoxicity pathway.

In accordance with previous findings showing a decrease in NAA associated with a reduced energy state [21], we found lower amounts of NAA in the ipsilateral thalamus. The behavior of glutamic acid was interesting; with a significant decrease in the ipsilateral thalamus in TBI mice compared to sham but not in the ipsi- and contralateral hemispheres, or whole brain. In contrast to our findings, a recent study by Sowers and colleagues [21] had previously identified with MSI higher levels of glutamic acid in the injured regions 24 h after lateral fluid percussion injury, a model characterized by a more diffuse injury compared to ours. This difference suggests that glutamic acid changes are time dependent with acute glutamate increase associated to excitotoxicity and a metabolic energy crisis. Whereas, 3 weeks after injury the reduction of glutamic acid in association to the decrease in NAA may point to a state of metabolic depression. It should also be acknowledged that the spatial and temporal changes of metabolites in the injured area and remote brain regions may be dependent on TBI model selection. In addition, it is important to note that a study conducted by Guerriero and colleagues [34] reported that a breakdown in the ratio of glutamic acid to GABA in the brain could be considered one of the mechanisms leading to post-traumatic epilepsy. While additional research is needed to determine whether changes in metabolite levels in the thalamus could contribute to the long-term consequences of TBI, this evidence may serve as an additional contribution for understanding the site-specific mechanisms underlying the enduring effects of TBI. Thus, data support the use of MSI approach in investigating ROI-based metabolic changes after experimental TBI. Future studies might be focused on more accurate LC-MS/MS studies, using a laser capture microdissection instrument, to accurately quantitate metabolite changes in selected regions.

## Conclusion

Understanding the molecular mechanisms underlying various diseases remains a significant challenge. Researchers have long sought methods to uncover metabolic changes in tissues.

We illustrate a novel MSI approach, incorporating a combined mass spectrometry approach involving LC-MS/MS on the whole brain and AP-MALDI MSI, enabling the visualization of spatial distributions of small metabolites directly on tissue slices. If the same TBI study had been done on homogenates of the contra- and ipsilateral hemispheres, we would not have been able to identify the brain area most affected by these changes.

Our results demonstrate distinct metabolite distribution patterns in TBI mice underscoring the utility of MSI as a valuable tool for advancing scientific research in this domain.

### Supplementary Information

Below is the link to the electronic supplementary material.

Supplementary file1 (DOCX 3.16 MB)
