# Supplementary material for: Mapping small metabolite changes after traumatic brain injury using AP-MALDI MSI
Source: Anal Bioanal Chem. 2024 Aug 1;416(22):4941–9. doi: 10.1007/s00216-024-05422-6 (PMC11330407; doi:10.1007/s00216-024-05422-6)

**Mapping Small Metabolite Changes After Traumatic Brain Injury using AP-MALDI MSI**

Angela Marika Siciliano^1^, Federico Moro^2^, Giulia De Simone^3^, Francesca Pischiutta^2^, Aurelia Morabito^3,4^, Roberta Pastorelli^3^, Laura Brunelli^3^, Elisa R. Zanier^2*^ and Enrico Davoli^1*^

^1^ Mass Spectrometry Research Center for Health and Environment and Laboratory of Mass Spectrometry, Environmental Health Sciences Department, Istituto di Ricerche Farmacologiche Mario Negri IRCCS, 20156 Milan, Italy

^2^ Laboratory of Traumatic Brain Injury and Neuroprotection, Department of Acute Brain and Cardiovascular Injury, Istituto di Ricerche Farmacologiche Mario Negri IRCCS, Milan, Ita

^3^ Laboratory of Protein and Metabolites in Translational Research, Environmental Health Sciences Department, Istituto di Ricerche Farmacologiche Mario Negri IRCCS, 20156 Milan, Italy

^4^ Department of Electronics, Information and Bioengineering, Politecnico di Milano, 20133 Milan, Italy

*Corresponding author: [enrico.davoli@marionegri.it](mailto:enrico.davoli@marionegri.it) Tel.: +39 0239014399

**Table S1** List of small metabolites identified using AP-MALDI-MSI targeted approach in positive ion mode

| Classes | Metabolite | *m/z* |
| --- | --- | --- |
| Nucleobases | Adenine | 136.0618 |
| Nucleobases | Guanine | 152.0567 |
| Nucleoside | Adenosine | 268.104 |
| Nucleoside | Inosine | 269.088 |
| Purine derivative | Hypoxanthine | 137.0458 |
| Neurotransmitter | GABA | 104.0706 |
| Neurotransmitter | Histamine | 112.0869 |
| Oxypurine | Uric Acid | 169.0356 |
| Amino acid | 4-Hydroxyproline | 132.0655 |
| Amino acid | 5-Oxyproline | 130.0499 |
| Amino acid | Alanine | 90.055 |
| Amino acid | Arginine | 175.119 |
| Amino acid | Aspartic Acid | 134.0448 |
| Amino acid | Glutamic Acid | 148.0604 |
| Amino acid | Glutamine | 147.0764 |
| Amino acid | Histidine | 156.0768 |
| Amino acid | Isoleucine | 132.1019 |
| Amino acid | Leucine | 132.1019 |
| Amino acid | Lysine | 147.1128 |
| Amino acid | N- Acetylaspartic Acid | 176.0553 |
| Amino acid | Phenylalanine | 166.0863 |
| Other | O-Phosphoethanolamine | 142.0264 |
| Sugar | Threonic Acid | 137.0445 |

**Table S2** Name, polarity, precursor Ion, target ion and confirmation ion of the analytes monitored in the Targeted metabolomic methods

| Name | Polarity | Precurson ion (*m/z*) | Target ion  (*m/z*) | Confirmation ion (*m/z*) | |
| --- | --- | --- | --- | --- | --- |
| Inosine | **+** | 269.1 | 137.2 | | 110.15 |
| Alanine | **+** | 79.9 | 44.15 | |  |
| Lysine | **+** | 147.2 | 84.1 | | 130.1 |
| Histidine | **+** | 155.9 | 110.1 | | 83.1 |

**Fig. S1** Spatial distribution of metabolites identified with AP-MALDI-MSI in sham and TBI mice brains; **a.** Metabolites identified in sham models; **b.** Metabolites belonging to the TBI model. Each line displays the distribution of the metabolite reported on the left in the six biological replicates. While some metabolites are uniformly distributed in the slices, others are not, allowing some anatomical regions to be distinguished.


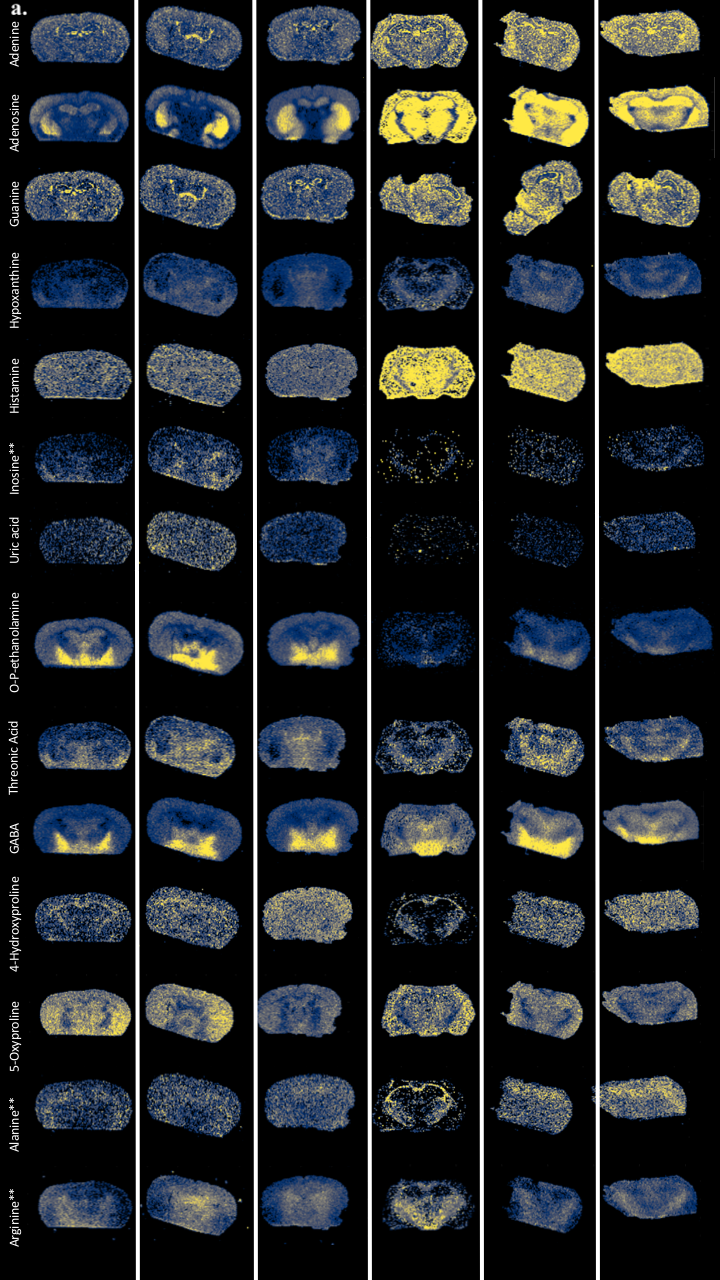

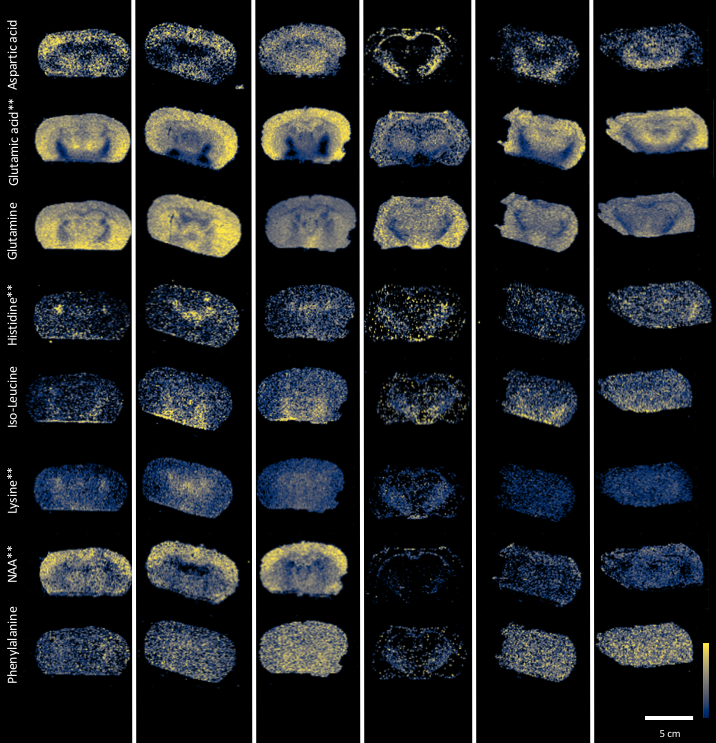

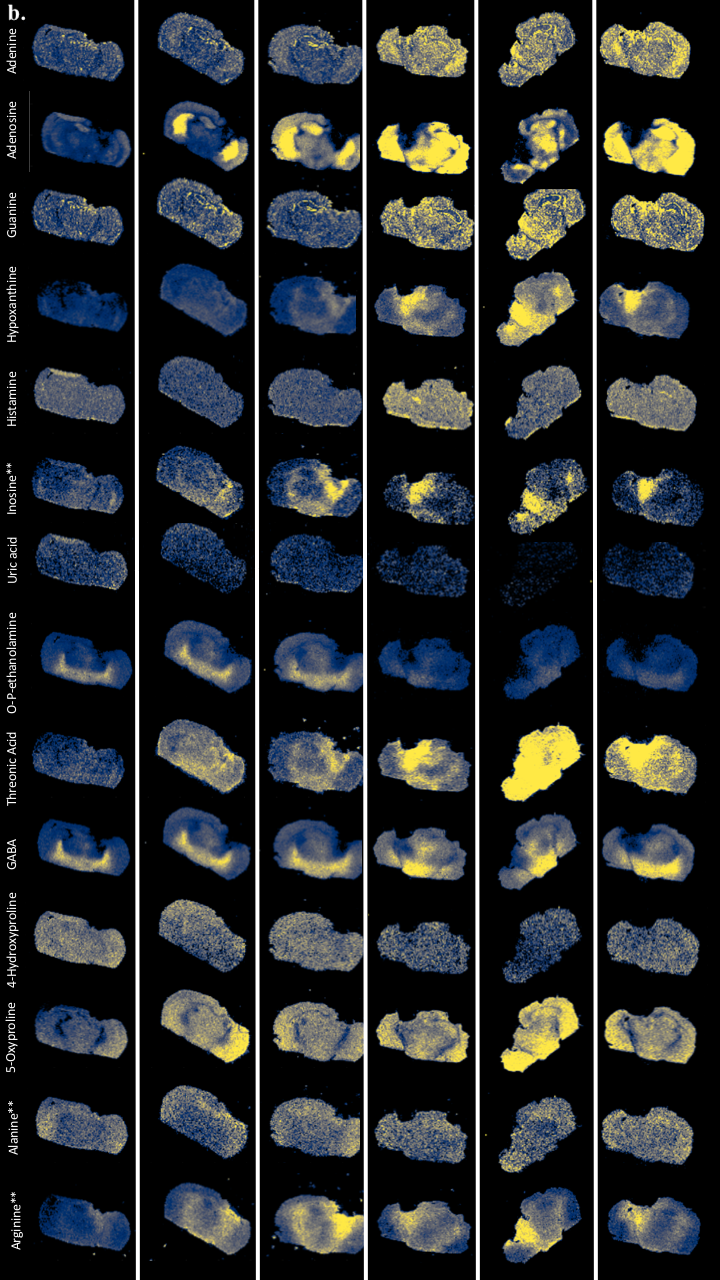

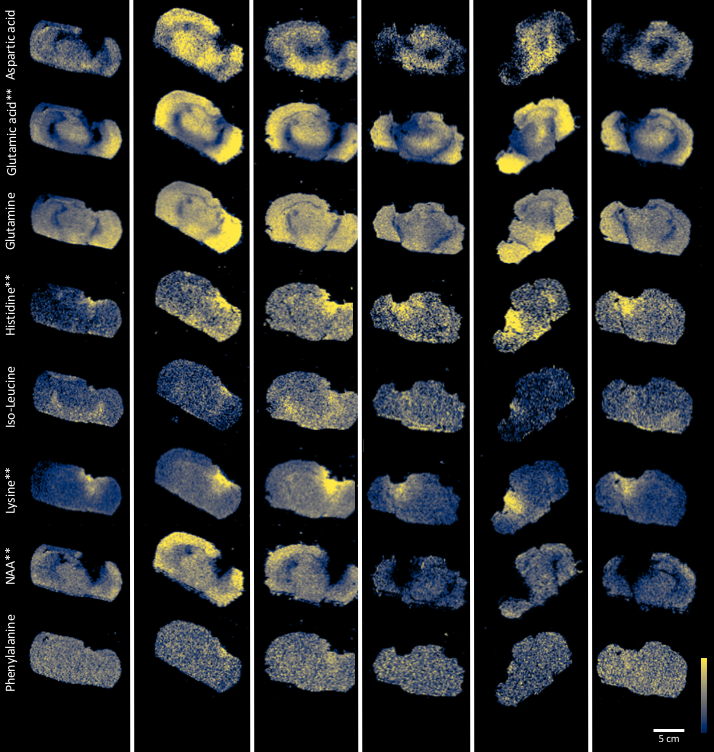

Supplement: Supplementary file 1 — Supplementary file1 (DOCX 3.16 MB) [file 216_2024_5422_MOESM1_ESM.docx]
